# Morphology of Neutrophils during Their Activation and NETosis: Atomic Force Microscopy Study

**DOI:** 10.3390/cells12172199

**Published:** 2023-09-02

**Authors:** Viktoria Sergunova, Vladimir Inozemtsev, Nina Vorobjeva, Elena Kozlova, Ekaterina Sherstyukova, Snezhanna Lyapunova, Aleksandr Chernysh

**Affiliations:** 1 Federal Research and Clinical Center of Intensive Care Medicine and Rehabilitology, V.A. Negovsky Research Institute of General Reanimatology, 107031 Moscow, Russia; va.inozemcev@physics.msu.ru (V.I.); waterlake@mail.ru (E.K.); kmanchenko@yandex.ru (E.S.); snezhanna.lyapunova@yandex.ru (S.L.); amchernysh@mail.ru (A.C.); 2 Koltzov Institute of Developmental Biology of Russian Academy of Sciences, 119334 Moscow, Russia; 3 Department of Immunology, Biology Faculty, Lomonosov Moscow State University, 119234 Moscow, Russia; nvvorobjeva@mail.ru; 4 Department of Medical and Biological Physics, Sechenov First Moscow State Medical University, 119991 Moscow, Russia

**Keywords:** neutrophils, AFM, CLSM, NET, nanostructures, membrane, nucleus, cell fragments, A23187, PMA

## Abstract

Confocal microscopy and fluorescence staining of cellular structures are commonly used to study neutrophil activation and NETosis. However, they do not reveal the specific characteristics of the neutrophil membrane surface, its nanostructure, and morphology. The aim of this study was to reveal the topography and nanosurface characteristics of neutrophils during activation and NETosis using atomic force microscopy (AFM). We showed the main stages of neutrophil activation and NETosis, which include control cell spreading, cell fragment formation, fusion of nuclear segments, membrane disruption, release of neutrophil extracellular traps (NETs), and final cell disintegration. Changes in neutrophil membrane nanosurface parameters during activation and NETosis were quantified. It was shown that with increasing activation time there was a decrease in the spectral intensity of the spatial periods. Exposure to the activator A23187 resulted in an increase in the number and average size of cell fragments over time. Exposure to the activators A23187 and PMA (phorbol 12-myristate 13-acetate) caused the same pattern of cell transformation from spherical cells with segmented nuclei to disrupted cells with NET release. A23187 induced NETosis earlier than PMA, but PMA resulted in more cells with NETosis at the end of the specified time interval (180 min). In our study, we used AFM as the main research tool. Confocal laser-scanning microscopy (CLSM) images are provided for identification and detailed analysis of the phenomena studied. In this way, we exploited the advantages of both techniques.

## 1. Introduction

Neutrophils are the most abundant white blood cells in the circulation and represent the first line of defense against pathogens. As professional phagocytes, neutrophils carry their antimicrobial weapons in granules and perform their effector functions such as phagocytosis, degranulation, and reactive oxygen species (ROS) generation at sites of inflammation. As was discovered by Arturo Zychlinsky and co-workers [1], neutrophils can also release neutrophil extracellular traps (NETs), which consist of decondensed chromatin decorated with antimicrobial proteins from granules, cytoplasm, and nucleus. The antimicrobial effect of NETs is due to the restriction of pathogen distribution or even their killing in situ. Predominantly, NET release results in the kind of cell death called NETosis [2]. In addition to their host defense function, NETs play an important role in the pathogenesis of autoimmune and inflammatory diseases [3,4,5,6,7,8]. NETs have also been implicated in thrombosis, various lung diseases, sepsis, and malignancy [5,6]. Understanding the signaling pathways that trigger NETosis is important to provide tools for treating patients.

The most commonly used pharmacological agonist of classical NETosis is phorbol 12-myristate 13-acetate (PMA). PMA activates protein kinase C (PKC) isoforms involved in the phosphorylation of the membrane-bound enzyme nicotinamide adenine dinucleotide phosphate (NADPH) oxidase, which is involved in the conversion of molecular oxygen to superoxide anion radicals ($O_{2}^{•-}$) [9].

The calcium ionophores A23187 and ionomycin promote NETosis, which occurs without the involvement of NADPH oxidase [10,11]. However, this form of NETosis still requires ROS, particularly mitochondrial (mtROS) [12] or those released by the pathogen itself [10]. Actin filaments [13,14,15], microtubules (MTs) [14,16], and lamins [14] are known to undergo significant changes during NETosis [13,17]. Morphological changes in cells undergoing NETosis were first published by Zychlinsky et al. in 2004 [1]. These results were obtained using different light microscopy methods. In 2018, Neubert E. et al. [14] performed a biophysical analysis of this process.

Most studies use electron (especially SEM) [18], and confocal microscopy and only a few studies have used atomic force microscopy (AFM) [19,20]. In our study, we used both confocal and AFM to take advantage of both techniques. AFM was used to study morphological changes in cells undergoing NETosis, and CLSM was used for characterizing, in parallel, these changes.

We show the features of neutrophil nanosurface topography and the transformation of internal cell structures during activation and NETosis. Two types of activators of different nature (A23187 and PMA) were used and the similarities and differences of their effects on neutrophils are shown.

## 2. Materials and Methods

### 2.1. Isolation of Primary Human Neutrophils

Venous blood from 5 healthy volunteers of both sexes, aged 25–40 years, was used in the study. Neutrophils were isolated according to the standard protocol for blood density gradient separation using Ficoll solutions [21].

All experiments involving blood were conducted in accordance with the Helsinki Declaration on Ethical Principles for Medical Research (2000) and the Protocol of the Council of Europe Convention on Human Rights and Biomedicine (1999) and were approved by the local ethics committee.

Briefly, the blood was separated on a double density gradient of Ficoll 1.119 g/cm^3^ and Ficoll 1.077 g/cm^3^ (Paneco, Russia) and centrifuged without brake (400× *g*, 40 min) using a Universal 320 centrifuge (Andreas Hettich GmbH & Co. KG, Tuttlingen, Germany). The neutrophil layer was then collected and washed three times in phosphate-buffered saline (PBS) without calcium and magnesium (MP Biomedicals, Illkirch, France).

Contaminating erythrocytes were lysed in cold hypotonic saline (0.2%) for 30 s, followed by reduction to physiological saline with hypertonic saline solution (1.6%). Isolated neutrophils were washed in phosphate-buffered saline (PBS) and suspended in complete medium consisting of RPMI 1640 supplemented with 10 mM HEPES, 2 mM L-glutamine, and 1% heat-inactivated fetal calf serum (FCS). Microscopic evaluation of the isolated cells showed that >97% were neutrophils. Cell viability was not less than 98% as determined by trypan blue exclusion. Neutrophils were stored at 4 °C (<1 h) prior to use in experiments.

### 2.2. Induction and Detection of Neutrophil Extracellular Traps

Freshly isolated neutrophils (2 × 10^5^ cells/mL) were allowed to adhere to sterile uncoated round coverslips placed in wells of a 24-well plate in complete medium (FCS, 1%) for 30 min at 37 °C, 5% CO_2_. NET formation was induced by 35 nM PMA (Sigma, St. Louis, MO, USA) or 2.5 µM A23187 (Sigma, St. Louis, MO, USA). NET formation was stopped at the indicated time points by adding 4% paraformaldehyde (PFA) (Sigma, St. Louis, MO, USA) to the well for 15 min. The coverslips were then carefully removed from the medium and washed 3 times for 5 min in PBS for the following procedures.

### 2.3. Atomic Force Microscopy

Images were obtained using an AFM “NTEGRA Prima” (NT-MDT Spectrum Instruments, Moscow, Zelenograd, Russia) in the tapping mode in air. Cantilevers NSG01 (NT-MDT Spectrum Instruments, Moscow, Zelenograd, Russia) were used with a tip radius of 10 nm, a resonance frequency of 87–230 kHz and a force constant of 5 N/m. The number of scan points was 512 or 1024 in each line of the image.

### 2.4. Membrane Nanostructure

To quantify the parameters of complex membrane configurations, we applied the spatial Fourier transform on 5 × 5 μm^2^ images and analyzed the spatial spectra [22]. For this purpose, the original specialized software FemtoScan Online Version 2.3.239(5.2) (Femtoscan, Moscow, Russia) was used.

The spatial spectrum is a function of the spectral intensity of $S_{i}$ (nm^2^) on the spatial frequency $v_{t_{i\;}I}$ = 1/$L_{i}$, where $L_{i}$ is the spatial period (nm). Spatial spectra can be calculated for different spectral windows, depending on the specific objectives of the study. In our study, the spectral windows were chosen according to the characteristic roughness of neutrophil membranes: 600–1200 nm (first order) for large sponge-like formations, 50–300 nm (second order) for small ones.

### 2.5. Fluorescence Staining of Neutrophils

For NETosis confirmation, fixed neutrophils were washed with PBS and permeabilized with 0.1% Triton X-100 in PBS for 2 min and RT. Nonspecific binding was reduced by preincubation of cells with blocking buffer (including human immunoglobulins) for 20 min. Immunofluorescence staining was performed with FITC-labeled monoclonal mouse anti-human MPO priming antibodies and neutrophil DNA was stained with DAPI (10 µM) for 10 min.

For CLSM and widefield microscopy, cells were permeabilized with TritonX-100 0.05% (Sigma, St. Louis, MO, USA) for 15 min, then washed and blocked with 3% BSA. DNA was stained with Hoechst 33,342 (Sigma, St. Louis, MO, USA) at 1:1000 for 20 min. Alexa Fluor 594-labeled wheat germ agglutinin (WGA) (Thermo Fisher Scientific, Waltham, MA, USA) (1:500 for 30 min in PBS) was used to stain neutrophil plasma membranes. The dyes were kept in the dark with the samples during staining. After staining, the coverslips were washed 3 times in PBS and then mounted on slides with Abberior Mount Solid Antifade (Abberior, Göttingen, Germany) before imaging.

### 2.6. Wide-Field Fluorescence Microscopy

Wide-field fluorescence microscopy was performed on a Thunder microscope (Leica Microsystems, Wetzlar, Germany ) equipped with LED excitation and a 63× oil immersion objective. Tile scans were performed on 8 basic fields for each coverslip. The mean number of cells was 150 ± 35. Image processing was performed using LAS X software version 3.0.0.15697 (Leica Microsystems, Wetzlar, Germany).

### 2.7. Confocal Laser-Scanning Microscope (CLSM)

A 405 nm laser was used to excite Hoechst 33342, a 488 nm laser was used to excite Alexa Fluor 488 phalloidin, and a 543 nm laser was used to excite Alexa Fluor 594 WGA. Images were captured using a Zeiss LSM880 (Carl Zeiss, Jena, Germany) confocal laser scanning microscope with a 63x immersion objective. Image processing, including conversion of the acquired z-stacks into maximum intensity projections (MIPs), was performed in Image J [23].

### 2.8. Statistical Analysis

#### 2.8.1. AFM

For each time point, 3 slides containing a monolayer of neutrophils were prepared. On each slide, 10 images were scanned at a size of 100 × 100 μm^2^ and from 50 × 50 μm^2^ to 30 × 30 μm^2^. The mean number of cells in a 100 × 100 μm^2^ image was 15 ± 8. To obtain data on structural features of neutrophil membranes and NETosis product, 5 × 5 μm^2^ and 1.5 × 1.5 μm^2^ regions were scanned on 10 cells for each sample. Statistical analysis of the results was performed using OriginPro 2019 software 9.8.0.200. (OriginLab Corporation, Northampton, MA, USA). Statistical data are presented as the mean with standard deviation (mean ± SD). The non-parametric Mann–Whitney test was used to test the significance of the difference between the data and the reference value. Differences were considered significant at * *p* < 0.05; ** *p* < 0.01; *** *p* < 0.001. Pearson’s correlation coefficient and regression coefficient were calculated.

#### 2.8.2. Wide-Field and Confocal Microscopy

One-way ANOVA with Bonferroni correction was used to assess differences between multiple groups. Data are expressed as mean ± SEM. Significant *p* values are indicated in the figures as follows: *, *p* < 0.05; **, *p* < 0.01; ***, *p* < 0.001.

Confocal microscopy was used to obtain z-stacks of 10 cells at each time point. These images were used to estimate nuclear volume. Nuclear volume data are presented as box plots. Using a wide field microscope, z-stacks of 8 fields were obtained at each time point. These images were used to evaluate cell size and cell number by group.

## 3. Results

**Figure 1:**
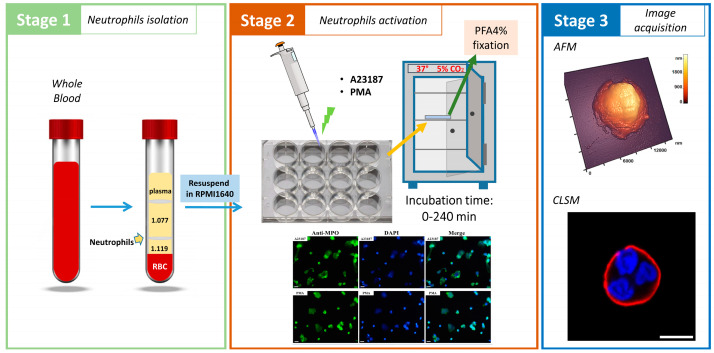
Scheme of experiment using human donor neutrophils exposed to A23187 and PMA. Stage 1: Isolation of neutrophils. Stage 2: Activation of neutrophils by A23187/PMA and exposure to CO_2_. Colocalization of decondensed chromatin with myeloperoxidase after neutrophil stimulation with PMA and A23187. Blue, staining of chromatin with DAPI; green, staining of MPO with FITC-conjugated anti-MPO monoclonal antibodies. Scale bar: 25 µm. Stage 3: Acquisition of AFM and fluorescence images.

Figure 1 shows the detailed scheme of the experiment.

In the experiment, neutrophils were first isolated using the two-step gradient technique [21] (Figure 1, stage 1). Neutrophils were scattered on glasses that were already in the wells of the plate. The sample plate was then placed in a ${\mathrm{CO}}_{2}$ incubator for 30 min. A23187 activator was then added to some of the wells and PMA activator to another part, while the others were left without activator (control) (Figure 1, stage 2). NETosis was confirmed by colocalization of chromatin with MPO in NET fibrils (Figure 1, stage 2). The samples were then fixed with 4% PFA at different stages of activation after 0, 30, 60, 120, 180 (PMA) and after 0, 30, 60, 120, 240 (A23187) minutes to obtain AFM images of the cells and fluorescence images. AFM images were obtained independently of fluorescence images (Figure 1, stage 3).

### 3.1. AFM Characterization of Neutrophils Morphological Changes upon Activation Recapitulated for Most of Those of CLSM

**Figure 2:**
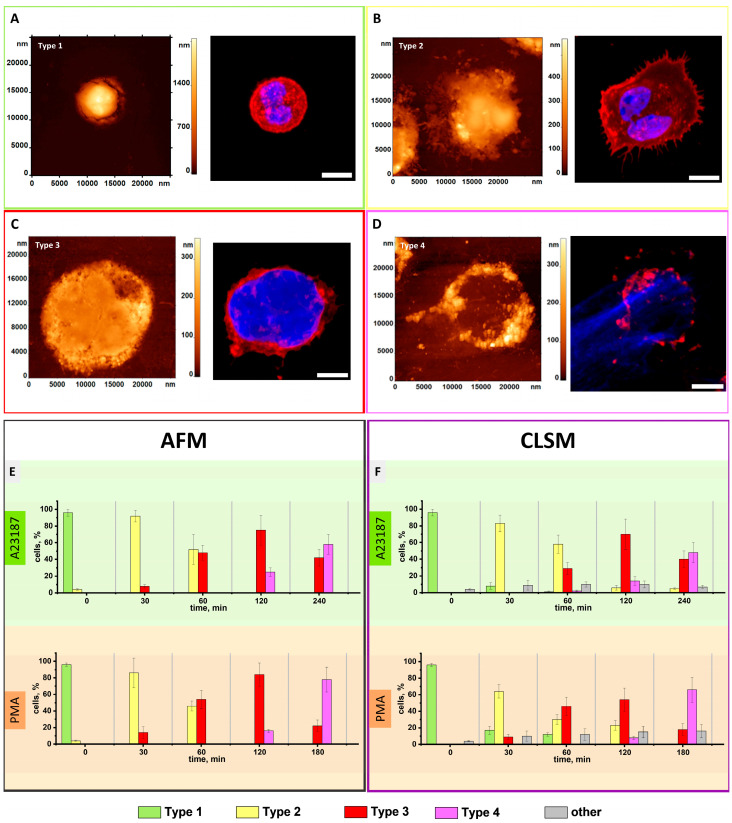
AFM 2D and CLSM images and statistical distribution of typical neutrophil types upon exposure to activators. (**A**) type 1; (**B**) type 2; (**C**) type 3; (**D**) type 4. (**E**) AFM histograms of the distribution of typical neutrophil types after exposure to A23187 and PMA, (N = 70 ± 12); (**F**) widefield microscopy histogram of the distribution of typical neutrophil types after exposure to A23187 and PMA, (N = 150 ± 35). Data are expressed as mean ± SD. The color of the box corresponds to the statistical group. In CLSM images, red color (membrane) is WGA + Alexa Fluor 594 dye, blue color of nuclear chromatin is Hoechst 33,342 dye. The scale of the confocal images is 5 μm.

The following four neutrophil types were identified from the obtained AFM and CLSM images: spherical cell (type 1, Figure 2A), spreading with segmented nucleus (type 2, Figure 2B), spreading with loss of nuclear segmentation (type 3, Figure 2C), and disrupted cells with NET release (type 4, Figure 2D). CLSM was used to confirm AFM observations.

In the non-activated state, the majority of neutrophils (96 ± 2%) from a healthy donor (control) had a spherical shape.

At 30 min after stimulation of neutrophils with the activator A23187, the majority of cells (92 ± 7%) had a type 2 shape (Figure 2B,E and Figure S1). At 60 min after activation, the percentage of type 2 cells decreased to 52 ± 18% and type 3 cells appeared (48 ± 9%). After 120 min, disrupted cells with NET (type 4, 25 ± 5%) began to appear (Figure 2D,E). The percentage of type 4 cells increased to 58 ± 12% after 240 min (Figure 2E). Notably, the percentage of type 1 cells was absent at 30 min. Correspondingly, there were absent type 2 cells left after 120 min of exposure.

After exposure of neutrophils to PMA for 30 min, the majority of cells (86 ± 14%) were type 2. Type 3 neutrophils appeared (14 ± 7%, Figure 2C), increasing to 54 ± 11% at 60 min. At 120 min after exposure, disrupted cells with NETosis appeared (type 4, 16 ± 2%) (Figure 2D,E). At 180 min, their percentage increased to 78 ± 15% (Figure 2).

The response of neutrophils to both activators shows a similar trend in the progression from type 2 to type 4 cells, representing a movement toward NETosis. However, there are differences in the timing and percentages at each stage, reflecting a distinct dynamic response to each activator. PMA appears to induce NETosis more rapidly, as evidenced by the higher percentage of type 4 cells at 180 min. In contrast, A23187 induces a more gradual progression.

The temporal changes in the percentage of type 1 cells were as follows: 96% at 0 min, 0% at 30 min, 0% at 60 min, 0% at 120 min. Correspondingly, type 2 cells were 46% at 60 min, whereas they were absent at 120–240 min of exposure.

We observed similar statistics in CLSM (Figure 2F). After 30 min of neutrophil stimulation with A23187, 83 ± 10% had a type 2, decreasing to 58 ± 11% at 60 min when type 3 and a small percentage of type 4 cells (2 ± 1%) appeared. Type 4 cells grew to 14 ± 5% at 120 min and 48 ± 12% at 240 min, with almost no type 2 cells remaining at that point (5 ± 1%). Type 1 cells were present at 8 ± 4% at 30 min and nearly absent at 60 min. With PMA, 64 ± 8% were type 2 at 30 min, and type 3 cells grew to 46 ± 11% at 60 min. Type 4 cells reached 8 ± 2% at 120 min and 66 ± 15% at 180 min. Different cells (approximately 10 ± 6%) were observed only in fluorescence images, which are labeled as “other” (Figure 2F).

**Figure 3:**
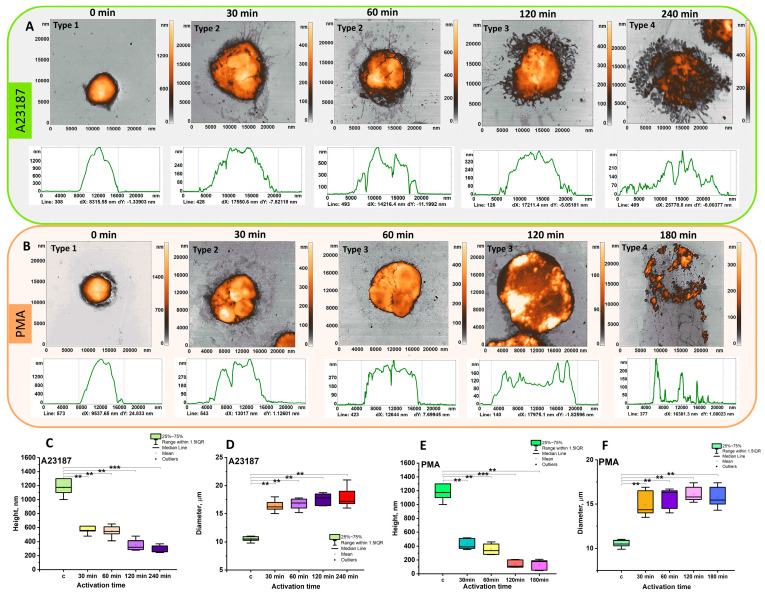
Changes in neutrophil shape (type 1→type 4) and size (height and diameter) upon exposure to activator. (**A**) AFM 2D images of neutrophils and their profile after exposure to A23187; (**B**) AFM 2D images of neutrophils and their height profile after exposure to PMA. Box plots of neutrophil heights (**C**,**E**) and diameters (**D**,**F**) as a function of exposure time to A23187 and PMA. N = 30. The 75th percentile and whiskers indicate mean standard deviation. Horizontal line indicates median and square indicates mean. ** *p* < 0.01; *** *p* < 0.001 (Mann–Whitney test).

Figure 3 shows a 2D AFM image of cells during their activation. A profile of heights at a given cross section is shown below each cell, with the cross-section line number indicated in each profile plot. The series of these figures shows the neutrophil transition in the following direction: type 1→type 2→type 3→type 4.

During the first 30 min of activation, the height decreased, and the diameter increased accordingly. At this time, spherocytic neutrophils (type 1, control) transformed into spherocytic neutrophils with a segmented nucleus (type 2).

Control neutrophils had a height of 1.2 ± 0.360 μm and a diameter of 9.8 ± 0.74 μm (Figure 3). When neutrophils were activated by A23187, cell height decreased approximately 2-fold compared to control during the first 30 min (Figure 3C) while the diameter increased approximately 1.7-fold (Figure 3D).

After 60 min, cell height was similar to 30 min (0.638 ± 0.114 μm) and cell diameter was unchanged from 30 min. Filopodia and cell fragments were formed around the cell.

At 120 and 240 (A23187) and 180 (PMA) minutes of activation, corresponding to the transition to type 3 and 4, cell height decreased approximately 4-fold compared to control. Cell diameter remained approximately the same as at 30 min (Figure 3C–F). The adhesion membrane appeared to contain “rod-shaped” structures when neutrophils were activated with A23187 (Figure 3A).

Similar changes in cell height and diameter were observed when neutrophils were activated with A23187 and PMA. Differences were observed in the structure of the adhesion membrane.

**Figure 4:**
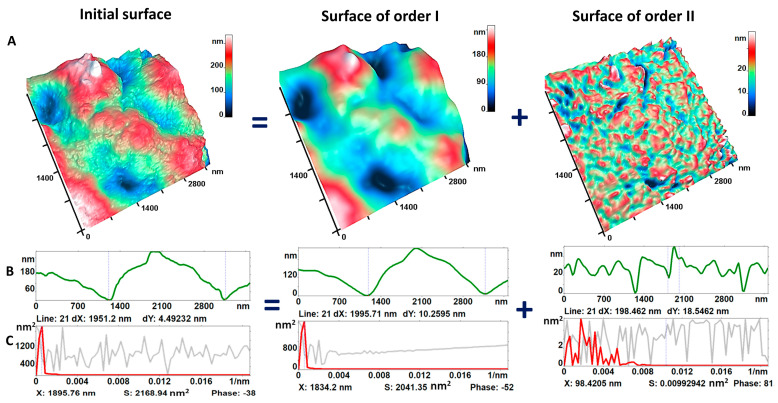

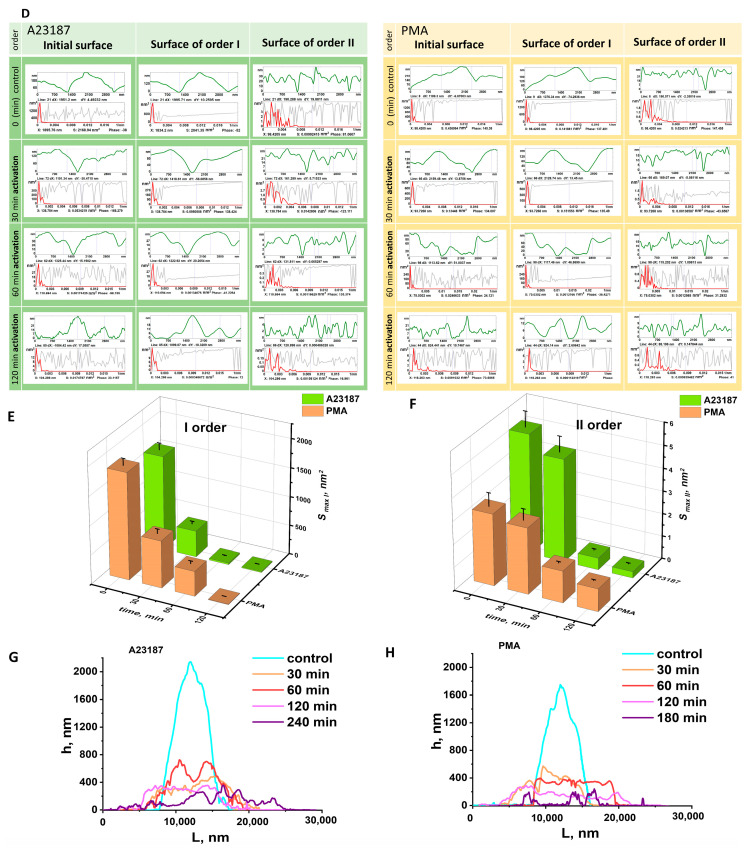
Changes in the nanostructure of neutrophil membranes. (**A**) AFM 3D images of the original surface and images of the first and second order surfaces. (**B**) Profiles (green plots) of the heights of the first and second order nanosurfaces of neutrophil membranes (control); (**C**) spatial spectrum of the heights of the corresponding membrane surfaces (red plots). (**D**) Fourier transform of the original neutrophil membrane profiles (green plots): Profiles (green plots) and corresponding spatial spectra (red plots) for the original surface, for the first order surface (spectral window 600–1200 nm), for the second order surface (spectral window 50–300 nm) for the activators A23187 and PMA; (**E**) histograms of the maximum intensity spectra of the first order surfaces $S_{I\;}\left(t_{i}\right)$ for the two activators; (**F**) histograms of the maximum intensity spectra of the second order surfaces $S_{II\;}\left(t_{i}\right)$ for the two activators; (**G**) plots of neutrophil profiles after exposure to A23187 and (**H**) after exposure to PMA.

Figure 3A,B shows the surface profiles of neutrophils for different activation times at different scales (1500 to 250 nm on the vertical scale). Although the cell height reduction cannot be clearly seen, the membrane surface characteristics and their changes during cell activation are shown in detail. The evolution of height reduction and neutrophil spreading along the surface is shown separately in Figure 4G,H.

Both AFM and CLSM were able to identify the four distinct neutrophil types. Temporal alterations in the percentage of various cell types in response to activators A23187 and PMA were consistently observed with both imaging modalities, such as the absence of type 1 and type 2 cells after specific exposure times. CLSM provides additional structural details that are not discernible through AFM, whereas AFM predominantly captures surface topography. AFM provides more detailed surface profile observations, which may not be captured by CLSM.

### 3.2. Nanosurface of Neutrophil Membranes during Activation

To measure the nanosurface parameters of the membranes, 5 × 5 μm^2^ regions were scanned on 10 cells for each sample and the spatial Fourier transform method was used as described in Section 2 [24,25]. The resolution for the nanostructure study was no more than 10 nm per pixel.

In the experiments, we investigated values *S_max_* и L_max_ [nm]. *S_max_* values characterize the roughness parameters of neutrophil membranes and, in general, the properties of their nanosurface. L_max_ [nm] indicates the size of the distances between the roughness maxima that occur at the intensity maximum. The intensity maxima indicate the values of the squares of the roughness amplitudes at a given spatial frequency.

Figure 4A shows an AFM image of the original membrane surface and an image of the first and second order surfaces. Figure 4B shows profiles (green plots) of the heights of the first and second order nanosurfaces of neutrophil membranes (control), and Figure 4C shows the corresponding spatial spectra (red plots).

Figure 4D shows the profiles of nanosurface heights of neutrophil membranes in a given cross-section (green plots) and the corresponding spatial spectra (red plots) for different activation times, for A23187 and PMA.

We refer to $S_{max\;t_{i}\;}$ [nm^2^] as the maximum intensity in the spatial spectrum of the first-order surface for a given activation time *t_i_*.

For the unactivated neutrophil, the maximum intensity spectra (*S_cont_*) were $S_{cont\;I\;}=1620\;\pm 120\;{\mathrm{nm}}^{2}$ and $S_{cont\;I\;}=1840\;\pm 120\;{\mathrm{nm}}^{2}$ for the first-order surface (Figure 4E). For the second order surface, $S_{cont\;II}$ was 5.3 ± 0.7 nm^2^ and $S_{cont\;II}$ was 3.2 ± 0.6 nm^2^ (Figure 4F). These values decreased during neutrophil activation.

Thus, for neutrophils after 30 min of exposure to A23187, $S_{30,\;I}$ was 450 ± 50 nm^2^ (Figure 4D,E) for the first-order surface and $S_{30,\;II}$ was 4.53 ± 0.7 nm^2^ for the second-order surface (Figure 4D,F). The trends of decreasing values of $S_{max\;t_{i}\;I}$ and $S_{max\;t_{i}\;II}$ are shown in Figure 4D–F. Similar changes in the reduction of $S_{max\;t_{i}\;I}$ and $S_{max\;t_{i}\;II}$ were observed for exposure to PMA activator, as shown in Figure 4D–F.

In the interval from 30 to 120 min, the $v_{maxt_{i\;}I}$ value after exposure to A23187 did not change much: for the first order, from 0.001 to 0.002, i.e., the characteristic spatial periods $L_{max\;t_{i}\;}$ decreased from 1000 nm (control) to 500 nm (120 min after activation).

For the second order, $v_{maxt_{i\;}II}$ varied between 0.0056 and 0.0049, i.e., the characteristic distance between the roughness peaks $L_{max\;t_{i}\;}$ varied between 200 nm and 110 nm during the whole activation time. The intensity of the $S_{i\;}$ spatial spectrum decreased dramatically with activation: from 15,600 nm^2^ in the control to 15 nm^2^ for 120 min after activation for the first order, and from 5 nm^2^ to 0.6 nm^2^ for the second order. Thus, the characteristic roughness dimensions were maintained in both the first and second orders as activation progressed, but the roughness heights decreased significantly: by a factor of 100 for the first order and by a factor of 8 for the second order. That is, the spongiform surface of the neutrophil became more frequent and had smaller amplitudes of peaks and troughs as activation progressed.

Upon exposure to PMA activator, similar changes in $v_{maxt_{i\;}I}$ were observed for the first-order surface. For the second order, the value of $v_{maxt_{i\;}II}$ varied insignificantly in the range of 0.006 to 0.008. For the whole activation time, the spatial roughness period $L_{max\;t_{i}}$ varied in the range of 125 to 166 nm. With increasing activation, the intensity of the $S_{i}$ spatial spectrum also decreased: from 1800 nm^2^ in the control to 10 nm^2^ for 120 min after activation for the first order, and from 3 nm^2^ to 1 nm^2^ for the second order. As activation progressed, the characteristic roughness sizes were maintained for both the first and second order, but the roughness heights decreased significantly: by a factor of 180 for the first order and by a factor of 3 for the second order.

In other words, the neutrophil nanosurface was stretched and straightened, becoming smoother as NETosis approached.

The height profiles shown in Figure 4G,H characterize the cell topology and demonstrate the evolution of cell spreading. The parameter $h_{max}$ is the maximum height of the cell (Figure 4G,H). The activators A23187 and PMA induce similar dynamics of $h_{max}$ height reduction in neutrophils. At 30 min after exposure to the activators, $h_{max}$ was 3- to 4-fold smaller than that of the control. At 120 min after activation, $h_{max}$ decreased a further 1.3–2.8-fold compared to 30 min.

A close correlation between the maximum cell height ($h_{max}$) and maximum intensity in the spectrum ($S_{max}$) was found for first and second order. For A23187, the Pearson correlation coefficient $r_{A23187\left(I\;order\right)}=0.94$ and $r_{A23187\left(II\;order\right)}=0.64$, while for PMA, the Pearson correlation coefficient $r_{PMA\left(I\;order\right)}=0.97$ and $r_{PMA\left(II\;order\right)}=0.77$.

Accordingly, the regression coefficient for the first-order A23187-activated neutrophil nanosurface $b_{A23187\left(I\;order\right)}=0.86\pm 0.23\;\mathrm{nm}$ and for the first-order PMA-activated neutrophil nanosurface $b_{PMA\left(I\;order\right)}=1.12\pm 0.21\;\mathrm{nm}$. Then the ratio $b_{A23187\left(I\;order\right)}/b_{PMA\left(I\;order\right)}=0.77$.

For the second order, the regression coefficient for the A23187-activated neutrophil nanosurface $b_{A23187\left(II\;order\right)}=0.002\pm 0.001\;\mathrm{nm}$ and for the PMA-activated neutrophil nanosurface $b_{PMA\left(II\;order\right)}=0.0010\pm 0.0007\;\mathrm{nm}$. Then the ratio $b_{A23187\left(II\;order\right)}/b_{PMA\left(II\;order\right)}=2$. These data demonstrate the different effects of these two activators on the neutrophil membrane nanosurface.

### 3.3. AFM Characterization of Cellular Transformation upon Activation

**Figure 5:**
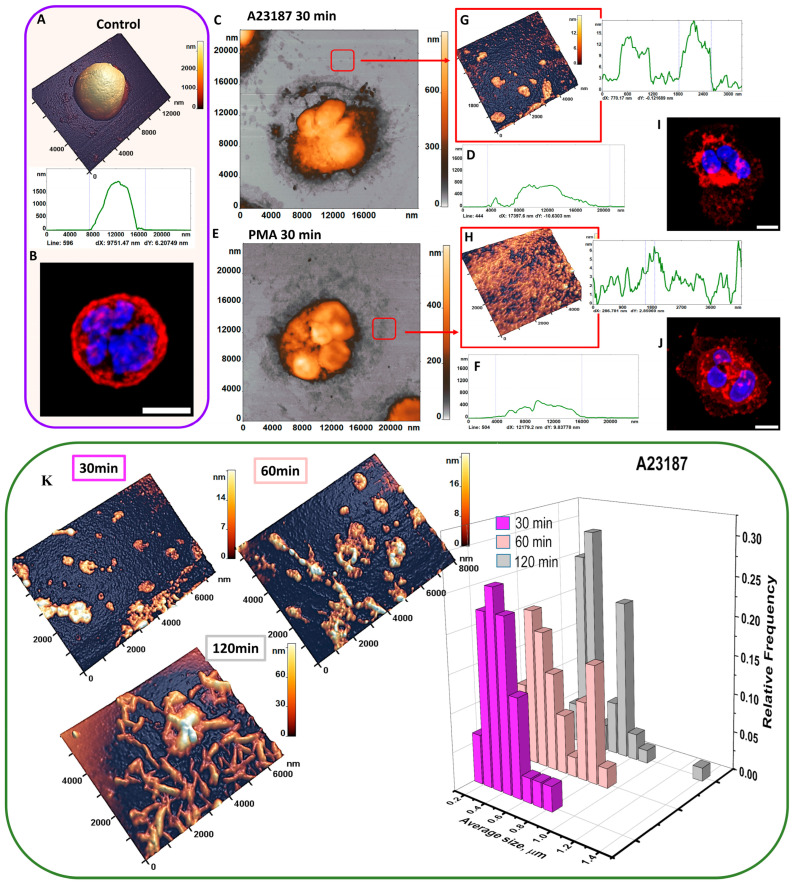
Changes in the structural configuration of neutrophils after exposure to the activators A23187 and PMA at 30 min. (**A**) AFM 3D image of control neutrophil and its profile; (**B**) CLSM image of control neutrophil; (**C**) AFM 2D image of neutrophil after stimulation with activator A23187 and its profile (**D**); (**E**) AFM 2D image of neutrophil after stimulation with activator PMA and its profile at 30 min (**F**); (**G**) 3 D image of a 5 × 5 μm^2^ area and its profile at 30 min after exposure to A23187; (**H**) 3D image of a 5 × 5 μm^2^ area and its profile at 30 min after exposure to PMA; (**I**) CLSM image of neutrophil after stimulation with A23187 at 30 min; (**J**) CLSM image of neutrophil after stimulation with PMA at 30 min. In CLSM images, red is WGA + Alexa Fluor 594, green is phalloidin + Alexa Fluor 488 and blue is Hoechst33342. Scale bar: 5 μm. (**K**) AFM 3D image of a 6 × 5 μm^2^ section after exposure to A23187 at 30 min, 60 min; 120 min, histograms for the average size of cell fragments “halo” after exposure to A23187 at 30, 60, 120 min.

Figure 5 shows the change in the structural configuration of neutrophils under the action of the activators A23187 and PMA after 30 min. AFM imaging can be used to study the shape of the cell and its nanosurface. Figure 5A shows the 3D AFM image of a control neutrophil and its profile. It had a maximum height of nearly 2 μm and a diameter of 9.5 μm. The cell was spherical in shape. In the CLSM image, the cell had a similar spherical shape. This image shows the membrane (red channel), and the segmented nucleus (blue channel) (Figure 5B).

AFM registration of the cell (Figure 5C,E) and its profile (Figure 5D,F) 30 min after exposure to both activators showed segmented nuclei, “loose” membrane structures and a membrane “halo” formed by cell fragments (Figure 5G for A23187). Cell fragments with an average size of less than 1 μm are present on the profile of the highlighted area. When neutrophils were exposed to PMA activator, structures ranging in size from 150 nm to 1 μm were absent in the highlighted area (Figure 5E).

CLSM showed (Figure 5I,J) that activated neutrophils changed shape after 30 min, from type 1 to type 2. A “halo” of adherent membrane was observed around the central part of the cell (Figure 5I,J).

#### 3.3.1. Cell Fragments

From the 60th minute, cell fragments began to be released from the cell, which is one of the components of activation. The cell fragments formed a kind of “halo” around the cell, which remained until the end of the observations. The number of cell fragments was counted on 3 identical 6 × 5 μm^2^ sections for each image. Upon exposure to A23187, the number of cell fragments increased with time, as did their average size. At 30 min, there were 26 ± 5 cell fragments (Figure 5K), at 60 min, there were 40 ± 7 cell fragments, and at 120 min, the number of cell fragments had increased to 58 ± 9.

Figure 5K shows histograms of the relative frequencies of the average size of cell fragments after exposure to A23187 for 30, 60, 120 min. The heights of these structures increased with time: from 16 ± 4 nm at 30 min to 61 ± 13 nm at 120 min.

At 30 min after exposure to A23187, the average size of the cell fragments was 0.33 ± 0.15 μm. In the following minutes, the average size of the cell fragments and their distribution increased. After 60 min, the average size of the cell fragments increased to 0.40 ± 0.19 μm. The statistical distribution of the average cell fragments size gradually changed from normal (30 min) to bimodal (120 min) over the activation time. At the end of this period, the histograms had two modes of 0.25 μm and 0.81 μm (Figure 5K).

The average size of cell fragments and membrane-attached structures for 30, 60, and 120 min did not differ significantly (*p* > 0.05).

The transformation of the distribution of the average size of cell fragments from unimodal to bimodal indicated that the size of cell fragments ejected from the cell increased with activation time and they fused to form larger structures.

#### 3.3.2. Characteristic Changes in the Nucleus Estimated by AFM

**Figure 6:**
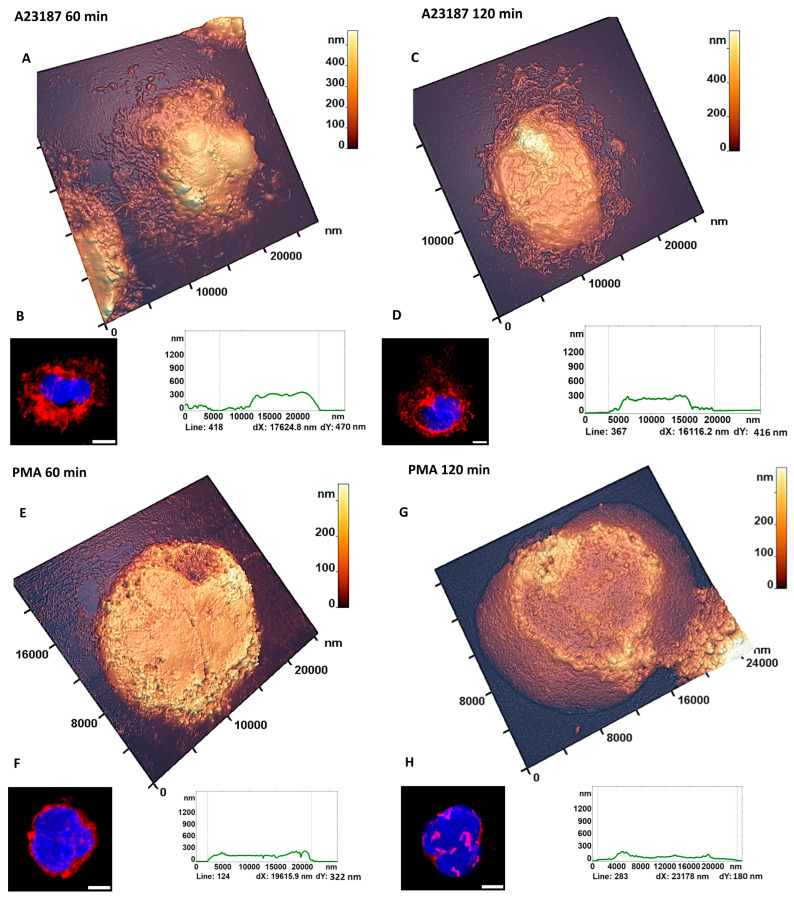
Characteristic changes in the structural configuration of neutrophils upon exposure to the activators A23187 and PMA at 60 and 120 min. (**A**) 3D AFM cell profile and (**B**) CLSM images of neutrophils at 60 min after exposure to A23187. (**C**) 3D AFM cell profile and (**D**) CLSM images of neutrophils 120 min after exposure to A23187. (**E**) 3D AFM cell profile and (**F**) CLSM images of neutrophils 60 min after exposure to PMA. (**G**) 3D AFM cell profile and (**H**) CLSM images of neutrophils 120 min after exposure to PMA. In CLSM images, red is WGA + Alexa Fluor 594, blue is Hoechst 33342. Scale bar: 5 µm.

When exposed to the activator A23187 for 60 min, nuclei did not increase in size and remained segmented in 58 ± 11% of the total neutrophils in the sample (Figure 2E and Figure 6A,B). After 120 min of activation, nuclei had lost segmentation and were completely dispersed throughout the cell volume up to the membrane contour (Figure 6C,D). Such cells represented 70 ± 18% of the total cell number (Figure 2E).

After PMA stimulation, nuclei were no longer segmented at 60 min (46 ± 11%) and filled the cell volume (Figure 2F and Figure 6E,F). At 120 min after activation, partial membrane disruption was observed (Figure 6G,H). The AFM image shows the structure of the central part of the cell, where the nucleus and part of the cellular structure beyond the cell membrane contour were located. The height of such a structure beyond the membrane contour is 26 ± 7 nm and the width is 4232 ± 1470 nm. The CLSM image shows a swollen nucleus occupying the entire cell volume 54 ± 14% of the cells, (Figure 2E,F and Figure 6H).

Thus, activation of neutrophils by exposure to the stimulants A23187 and PMA leads to different changes in cell membrane structure.

Neutrophil activation did not result in NETosis in all cases. A fraction of cells maintained membrane integrity after exposure to A23187 and PMA and did not release NETs, although the processes of nuclear segment fusion and chromatin decondensation were underway. Under the conditions of our experiments, neutrophils that did not undergo NETs amounted to approximately 20–25% of the total number of control cells.

#### 3.3.3. NETosis

**Figure 7:**
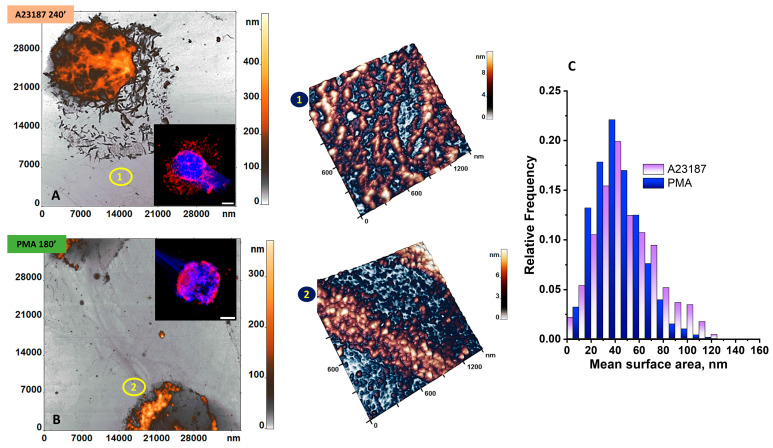
Characteristic 2D AFM and CLSM images of neutrophil NETosis at 240 min upon exposure to the activator A23187 (**A**) and at 180 min upon exposure to the activator PMA (**B**). (**1**) 3D AFM image of NETosis results in a 1.5 × 1.5 μm^2^ area after exposure to activator A23187; (**2**) 3D AFM image of NETosis results in a 1.5 × 1.5 μm^2^ area after exposure to activator PMA. (**C**) Histograms for the mean surface area of granules after exposure to A23187 purple and PMA blue. Total granules: A23187-468, PMA-820. Mean size, nm: A23187, 50 ± 20, PMA, 30 ± 10. Scale bar: 5 μm.

Neutrophil NETosis in our experiments was observed between 180 min for PMA and 240 min for A23187 and is shown in AFM and CLSM images in Figure 7.

Figure 7 shows the initial development of NETosis: the membrane begins to degrade and granular fragments are seen in the extracellular space. Nuclear material with the cytoplasmic contents of the cell leaked out of the cell as mesh-like structures (Figure 7A(1),B) consisting of granules. The integrity of the cell and its membrane was disrupted.

In addition, “rod-shaped” membrane fragments were retained during A23187-stimulated and the membrane was generally not lysed (Figure 7A).

The total number of granules was 2-fold lower after exposure to A23187 than after exposure to PMA. The mean surface area granule size was essentially unchanged and was 50 ± 20 nm for A23187 and 30 ± 10 nm for PMA. All mean surface area granule sizes for A23187 and PMA did not differ at *p* > 0.05 (Figure 7C). However, the mean surface area size of granules (1) and (2) was significantly smaller than the “cell fragments halo” (Figure 5K) size by approximately 6–10 times. Neutrophil NETosis in our experiments was observed between 180 min for PMA and 240 min for A23187 and is shown in AFM and CLSM images in Figure 7.

AFM imaging of NETs is a challenging task. Once expelled into the extracellular environment, DNA networks disperse due to diffusion currents and their density decreases sharply. In addition, the structure of the networks is severely damaged by the action of fixatives used in AFM studies.

## 4. Discussion

The results presented above provide an in-depth analysis of neutrophil morphological changes upon activation, using two imaging techniques: AFM and CLSM. Neutrophil activation, especially NETosis has been a subject of intense research in recent years, particularly its role in immune response [7,19]. Existing literature on NETosis often focuses on molecular and cellular aspects like chromatin decondensation and cytoskeletal rearrangements [20,26,27,28,29,30].

In our work, we distinguished four different types of neutrophils and their progressive changes upon exposure to two activators, A23187 and PMA. This classification highlights distinct morphological states, which are recognized in the literature [31]. The identification of stages from control cell to final disintegration of the cell is consistent with what is generally known about NETosis but the AFM results add a level of specificity. Morphological studies of neutrophils have been carried out previously by other authors [32,33], but the observation of “rod-shaped” structures in the adhesion membrane and the intricate details of cell surface alterations add to the field’s understanding of neutrophil morphology.

The application of AFM for studying biological membranes is well established [22,24,34,35]. The spatial Fourier transform was used to transform a surface into a spatial spectrum. The obtained spectra were used to estimate parameters such as spectral intensity *S_max_* and spatial period *L_max_* which characterized the roughness of neutrophil membranes. The first-order spatial spectrum for both activators was narrow and remained essentially unchanged with activation. That is, the spongiform structure of neutrophils was initially regular and stable during the first 120 min of activation. The second-order spectrum in controls was significantly broader and more stochastic. Thus, as activation progressed, the membrane surface became more chaotic and variable at the nanoscale. Using AFM, we were able to show that as the activation time increased, the spectral intensity of the spatial periods decreased. In the early stages of NETosis, the surface of neutrophils stretched and became smooth; $S_{\max}$ decreased to 5–10 nm. Such phenomenon was not observed in confocal microscopy. Spectral characteristics for the first and second order surfaces were measured during 120 min after activation. For longer time periods, such registration was not meaningful because membrane destruction began, and its integrity was compromised. In existing literature [36], the complexity of membrane organization at different scales is recognized but not often characterized in such specific terms.

AFM registration revealed segmented nuclei and “loose” membrane structures, with a specific membrane “halo” (Figure 5G). Starting from the 60th minute, cell fragments began to be released, forming a kind of “halo” around the cell, which is one of the components of activation [37]. This “halo” phenomenon may be related to the process of vesiculation, a known stage in neutrophil activation [38,39]. Upon exposure to A23187, the quantity and average size of cell fragments increased over time (Figure 5K). This is consistent with studies that demonstrate changes in cell morphology during activation [37]. The transformation of the distribution of the average size of cell fragments from unimodal to bimodal indicates an increase in the number and in the size of cell fragments ejected from the cell and their fusion to form larger structures. This could be due to the activation of µ-calpain and ezrin proteins during NETosis, leading to membrane permeabilization [40]. This resulted in a rapid disassembly of the actin cytoskeleton, followed by the shedding of plasma membrane microvesicles [38].

After 60 min of A23187 exposure nuclei remain segmented in a significant number of neutrophils but lose segmentation after 120 min. In contrast, PMA stimulation seems to induce more rapid nuclear changes. This gradual change over time may reflect a specific pathway or mechanism of activation. In the literature, A23187 is known as a calcium ionophore, and its effect might be correlated with calcium-dependent signaling pathways within the cell. Additionally, PMA is known as a potent activator of protein kinase C (PKC), which is involved in various cellular signaling pathways [14,41,42,43].

NETs were visualized via AFM as a branching structure with filaments that branched and merged. Meshwork filaments consisting of granules 30–50 nm in size were identified (Figure 7), a phenomenon also observed in recent studies of NET structure [44]. Such granules were found at all sites where fluorescent DNA signal was detected.

Thus, in our study, we provided identification, detailed analysis, and characterization of the stages of NETosis activated by the classical activator PMA and calcium ionophore A23187 using AFM and CLSM. At the same time, many features characteristic of other forms of programmed cell death (PCD) (apoptosis, necroptosis, secondary necrosis, pyroptosis, autophagy) are also inherent in NETosis. In NETosis, as in apoptosis, coordinated changes occur in the nucleus and in the cytoplasm, although the nature of the changes is different, and NETosis, unlike apoptosis, does not require caspase activation. However, unlike apoptosis, NETosis (as well as other forms of PCD) is accompanied by disturbance of the insulating properties of the plasma membrane, while the mechanisms of permeabilization in NETosis, pyroptosis, and necroptosis are similar. The pathways leading to NETosis and necroptosis are especially strongly intertwined [45,46]. We hope that further investigations on NETosis and other forms of PCD will shed light on the interweaving of these sophisticated processes.

## 5. Conclusions

In our study, we used AFM to investigate the nanosurface parameters of neutrophil membranes during activation and NETosis. These parameters could not be obtained by conventional fluorescence microscopy methods. The results obtained may improve the understanding of immunological processes and can be used in the future for a more detailed study of the biophysical mechanisms of neutrophil activation and NETosis under different conditions.

## Data Availability

The datasets used and analyzed during the current study are available from the corresponding authors upon request.

## Supplementary Materials

The following supporting information can be downloaded at: https://www.mdpi.com/article/10.3390/cells12172199/s1, Figure S1: AFM and widefield fluorescence images after A23187 (time points 30′ and 240′) and PMA (time points 30′ and 180′) activation.
