# Supplementary material for: Morphology of Neutrophils during Their Activation and NETosis: Atomic Force Microscopy Study
Source: Cells. 2023 Sep 2;12(17):2199. doi: 10.3390/cells12172199 (PMC10486724; doi:10.3390/cells12172199)
Supplement: Supplementary file 1 [file cells-12-02199-s001.zip › cells-2561227-supplementary.pdf]

cells

MDPI

Article

Morphology of Neutrophils during Their Activation and NETosis: Atomic Force Microscopy Study

Viktoria Sergunova [1,*], Vladimir Inozemtsev [1,2], Nina Vorobjeva [3], Elena Kozlova [1,4], Ekaterina Sherstyukova [1,4], Snezhanna Lyapunova [1] and Aleksandr Chernysh [1]

[1] Federal Research and Clinical Center of Intensive Care Medicine and Rehabilitology, V.A. Negovsky Research Institute of General Reanimatology, 107031 Moscow, Russia; va.inozemcev@physics.msu.ru (V.I.); waterlake@mail.ru (E.K.); kmanchenko@yandex.ru (E.S.); snezhanna.lyapunova@yandex.ru (S.L.); amchernysh@mail.ru (A.C.)

[2] Koltzov Institute of Developmental Biology of Russian Academy of Sciences, 119334 Moscow, Russia

[3] Department of Immunology, Biology Faculty, Lomonosov Moscow State University, 119234 Moscow, Russia; nvvorobjeva@mail.ru

[4] Department of Medical and Biological Physics, Sechenov First Moscow State Medical University, 119991 Moscow, Russia

* Correspondence: vika_23s82@mail.ru

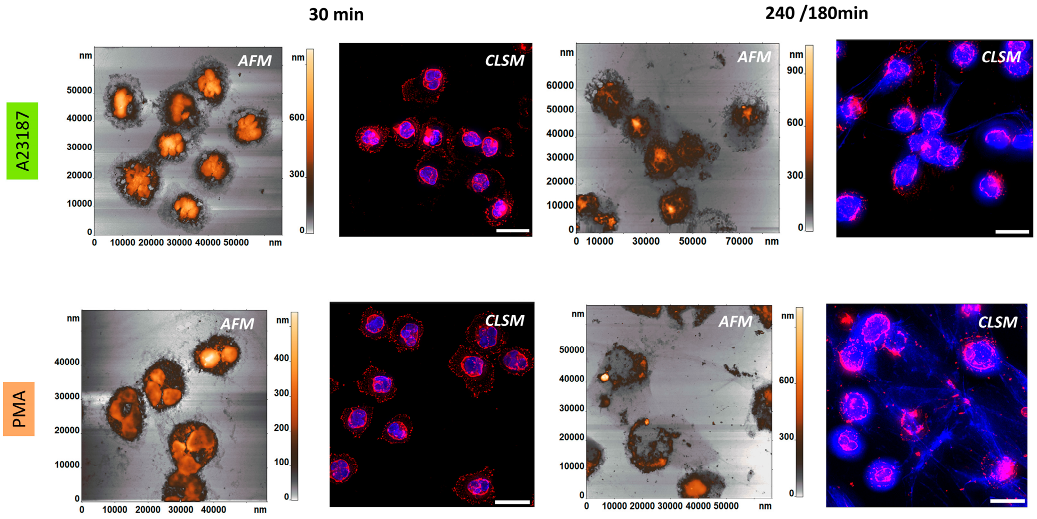

Figure S1. AFM and widefield fluorescence images after A23187 (time points 30′ and 240′) and PMA (time points 30′ and 180′) activation.
